# Long-term outcomes of imatinib in patients with FIP1L1/PDGFRA associated chronic eosinophilic leukemia: experience of a single center in China

**DOI:** 10.18632/oncotarget.8906

**Published:** 2016-04-21

**Authors:** Shi-Qiang Qu, Tie-Jun Qin, Ze-Feng Xu, Yue Zhang, Xiao-Fei Ai, Bing Li, Hong-Li Zhang, Li-Wei Fang, Li-Juan Pan, Nai-Bo Hu, Zhi-Jian Xiao

**Affiliations:** ^1^ MDS and MPN Centre, Institute of Hematology and Blood Diseases Hospital, Chinese Academy of Medical Sciences & Peking Union Medical College, Tianjin, China; ^2^ State Key Laboratory of Experimental Hematology, Institute of Hematology and Blood Diseases Hospital, Chinese Academy of Medical Sciences & Peking Union Medical College, Tianjin, China; ^3^ Molecular Diagnostic Laboratory, Institute of Hematology and Blood Diseases Hospital, Chinese Academy of Medical Sciences & Peking Union Medical College, Tianjin, China

**Keywords:** eosinophilia, FIP1L1-PDGFRA, chronic eosinophilic leukemia, imatinib

## Abstract

**Background:**

The FIP1L1/PDGFRA (F/P) fusion gene is the most common clonal genetic abnormality of chronic eosinophilic leukemia (CEL). Tyrosine kinase inhibitors (TKI), such as imatinib, have been demonstrated to be effective therapies for F/P mutated disease. The aim of this study was to analyze the treatment response and long term prognosis in patients with F/P mutated CEL.

**Methods:**

The clinical features and treatment responses of 33 consecutive patients with F/P mutated CEL between August 2006 and October 2014 were analyzed. The 33 cases received imatinib therapy at an initial dose of 100 mg/day (30 patients) or 200 mg/day (3 patients); the maintenance dose depended on the response condition and patient willingness. Through the follow up, the molecular responses were regularly monitored.

**Results:**

With a median follow up of 64 months, 94% of the 33 patients with F/P mutated CEL achieved a complete hematologic remission (CHR), and 97% achieved a complete molecular remission (CMR) after a median of 3 (1.5-12) months. Twenty-four cases received maintenance therapy, with a median CMR duration of 43 (5-88) months. Imatinib therapy was discontinued in 8 cases, including 4 cases who experienced relapse, and 4 patients who maintained CHR or CMR after discontinuing therapy with a median time of 47 (2-74) months. One case exhibited primary resistance with a PDGFRA T674I mutation.

**Conclusions:**

F/P mutated CEL has an excellent long-term prognosis following imatinib therapy. A 100 mg daily dose of imatinib is sufficient to induce remission, and a single 100 mg weekly dose maintains a durable remission. A subgroup of patients may maintain a durable remission after discontinuing therapy with a CMR.

## INTRODUCTION

FIP1L1/PDGFRA (F/P) rearrangement is the most common molecular abnormality in chronic eosinophilic leukemia (CEL). Two study groups [1, 2] in 2003 reported their independent discoveries of an F/P fusion gene that results from a cryptic deletion on chromosome 4q12 in patients with hypereosinophilic syndrome (HES); these patients were subsequently re-diagnosed with CEL. According to the specific molecular abnormality, the revised WHO classification in 2008 recognized the myeloid and lymphoid neoplasms with eosinophilia and abnormalities of platelet-derived growth factor receptor (PDGFR) A/B or fibroblast growth factor1 (FGFR1) as a new subgroup of myeloid neoplasms, which comprise three rare specific disease groups [3]. These 3 diseases result from a fusion gene that encodes an aberrant tyrosine kinase.

The tyrosine kinase inhibitor (TKI) Imatinib mesylate has revolutionized the therapy for PDGFR-related disease [4–12]. F/P kinase activity is more sensitive to imatinib than BCR-ABL, and the occurrence of drug resistance is rare [5]. The therapeutic effects have been established from previous reports [4–12]; a low dose (100-200 mg/day) obtained an excellent response, and a lower dose (100-200 mg/week) effectively maintained remission. Nevertheless, the long term prognosis, drug withdrawal and drug resistance therapy remain unclear. In this study, we retrospectively analyzed the treatment response and long term prognosis of 33 Chinese patients with F/P mutated CEL.

## RESULTS

### Patient characteristics

The laboratory characteristics of the patients with F/P mutated CEL are shown in Table 1. At presentation, most patients had severe eosinophilia (AEC≥5×10^9^/L) and a significantly increased serum vitamin B12 level. A significant proportion presented with anemia, thrombocytopenia, and myelofibrosis. The most frequent organ involvement of the F/P mutated disease occurred in the spleen (68%), lung (44%), skin (28%), liver (23%), and heart (22%). The constitutional symptoms, including fatigue, night swear, and weight loss, were frequent in the patients with F/P fusion. Thromboembolism was one of the most critical complications at presentation. One patient presented with thrombosis, and a digital subtraction angiography of the lower extremities for the left lower leg indicated a completely occluded thrombus of the left popliteal vein and collateral angiogenesis. Another patient presented with palpitation and dyspnea, and an echocardiography indicated a right ventricular mural thrombus.

**Table 1:** F/P (+) CEL group characteristics

Parameter	F/P(+)CEL
Number of patients	33
Male/Female(n)	33/0
Median age (range) (years)	35 (18-73)
WBC (×10^9^/L) (range)	39.1(3.86-210)
WBC≥30×10^9^/L, n(%)	15(44%)
Hb (g/L) (range)	117.6(71-168)
Hb<110 g/L, n(%)	12(35%)
BPC (×10^9^/L) (range)	137.5(16-422)
BPC<100×10^9^/L, n(%)	11(33%)
AEC (×10^9^/L) (range)	17(1.6-78.8)
AEC≥5×10^9^/L, n(%)	27(82%)
Eosinophils in bone marrow(%)	36(8-69)
Myelofibrosis (n)	13/30
Serum IgE(IU/mL)(range)	27.2(1-3660)
IgE>N*(n)	2/22
Serum B12(pmol/L) (range)	2180(262-2959)
Serum B12>N*(n)	13/19

WBC: white blood cell, Hb: hemoglobin, BPC: blood platelet count, AEC: absolute eosinophil count, IgE: immunoglobulin E,

* Normal ranges (N): IgE <165.3 IU/mL, vitamin B12 <800 pmol/L.

### Treatment response

**Figure 1:**
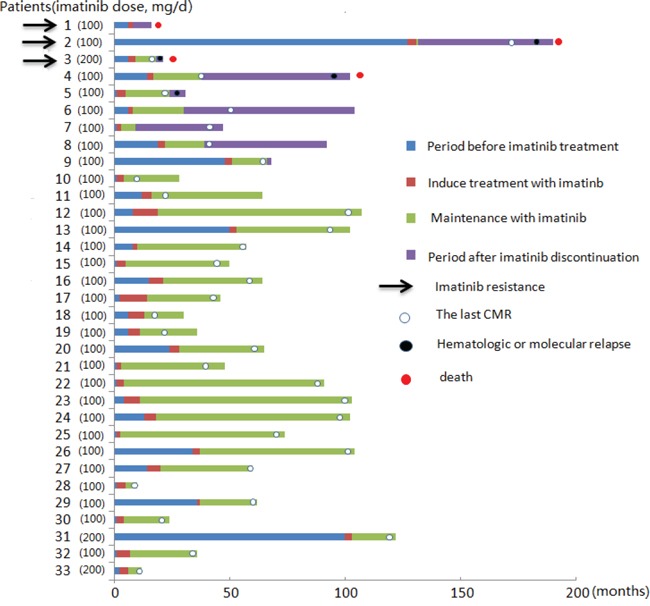
Imatinib therapeutic process and prognosis in patients with F/P(+) CEL

The median disease history was 6 (1-127) months prior to receiving imatinib treatment, and the median time of imatinib treatment was 30 (2-99) months. Thirty-one patients (94%) achieved a CHR, 1 patient achieved a PHR, and one patient had no hematological remission. The molecular response of the patients was evaluated regularly (Figure 1). Thirty-two patients (97%) achieved a CMR after a median of 3 (1.5-12) months.

With a median follow-up of 46 (9-100) months, 24 patients who achieved a CMR remained on maintenance therapy. The imatinib maintenance dose was 100 mg/day (patients 23 to 33), 100 mg/week (patients 11 to 19), 100 mg thrice weekly (patients 20 to 22), and 50 mg/week (patient 10). Two patients experienced a hematologic relapse; these patients had maintenance doses of 100 mg/week (patient 11) and 50 mg/week (patient 10), respectively. Two patients with a 100 mg/week dose remained in a CHR (patients 18 and 19) and were not monitored for fusion gene at the last contact. The remaining 20 patients remained in a CMR with a median duration of 43 (5-88) months.

### Cessation of imatinib mesylate

Eight patients (patients 2 to 9) discontinued imatinib treatment after they acquired CMR because of a lack of compliance (n =7) and medical decisions for reproduction (n = 1). Four patients (patients 2 to 5) experienced a hematologic relapse from 2 to 48 months after the discontinuation of imatinib. Two patients (patients 2 and 3) exhibited secondary imatinib resistance and altered other treatment. One patient (patient 4) died after relapse; however, the cause of death was unknown. One patient (patient 5) did not receive treatment because of the lack of symptoms. The other 4 patients (patients 6 to 9) remained in a CHR for a median of 47 months (range 2-74) after discontinuation; 2 patients (patients 7 and 9) maintained a CMR, and the other 2 patients (patients 6 and 8) were not monitored for F/P fusion gene for an interval of 3 months at the last contact (Table 2).

**Table 2:** Outcome of imatinib discontinuation in 8 patients

Patient (No)	Age	Initial dose	Months to CMR	Months of treatment duration	Months of imatinib interruption
2*	43	100 mg/d	4	4.5	48
3*	28	200 mg/d	3	12	2
4*	38	100 mg/d	3	24	12
5*	34	100 mg/d	4	18	5
6	73	100 mg/d	2	24	74
7	41	100 mg/d	2	8	39
8	32	100 mg/d	3	20	55
9	32	100 mg/d	3	18	2

* Hematologic relapse

### Imatinib-resistant therapy

Three patients (patients 1 to 3) exhibited imatinib resistance during the period of treatment (Table 3). One patient (patient 1) exhibited primary resistance with a PDGFRA T674I mutation; he subsequently received nilotinib treatment, but, unfortunately, a response was not achieved, and he died of cardiac complications. The other 2 patients exhibited secondary resistance 2 and 48 months after the discontinuation of imatinib. One patient (patient 3) was determined to have a T674I resistance mutation in the blast phase. He subsequently received imatinib 400 mg/day, and a combined homoharringtonine plus cytarabine regiment was implemented as a salvage treatment; however, the disease still progressed, and he died of multiple organ failure 41 days after relapse. The remaining patient (patient 2) experienced a relapse 48 months after cessation of imatinib with a T674I resistance mutation, and he successively received high-dose imatinib, nilotinib, dasatinib and COP (Cyclophosphamide, Oncovin, and Prednisone) regiments; however, the salvage treatments were not successful, and he died of cardiac failure 17 months after the molecular relapse.

**Table 3:** Clinical characteristics of the 3 patients with imatinib-resistance

Patient (No)	Patient 1	Patient 2	Patient 3
Age	57	43	28
Clinical manifestations	fatigue, weight loss, bone pain, splenomegaly	night sweats, fatigue, splenomegaly	cough, bone pain, splenomegaly
AEC at first diagnosis	7×10^9^/L	7×10^9^/L	4.32×10^9^/L
Initial karyotype	47, XY, +8[12]	46, XY[15]	46, XY[20]
Dose of imatinib (mg/day)	100→400	100	200
Time to imatinib resistance (months)	primary resistance	52.5	14
Mutation	T674I	T674I	T674I
Disease phase at diagnosis of resistance	CP	CP	BP
Therapy following resistance	nilotinib(400 mg BID), hydroxyurea(1g BID)	imatinib(100→400 mg/day), nilotinib(400 mg BID), dasatinib(100 mg/day), cyclophosphamide/oncovin/prednisone	imatinib 400 mg/day, homoharringtonine/cytarabine
Follow up after resistance	death after 14 months	death after 17 months	death after 41 days

CP: chronic phase, BP: blast phase, BID: twice a day.

### Side effects of imatinib

Imatinib was well tolerated with a low incidence of adverse events. Of the 33 cases, only 3 cases reported hematological toxicities, which were clearly associated with imatinib, and these events occurred at the initiation of therapy. One patient who was initially treated with a dose of 200 mg/day suffered grade III neutropenia and thrombocytopenia and was subsequently managed by a temporary discontinuation of imatinib. The other 2 patients who received imatinib at a dose of 100 mg/day suffered grade II neutropenia, and the neutrophil count recovered to normal with a decrease in the dose to 100 mg every other day.

### Survival

**Figure 2:**
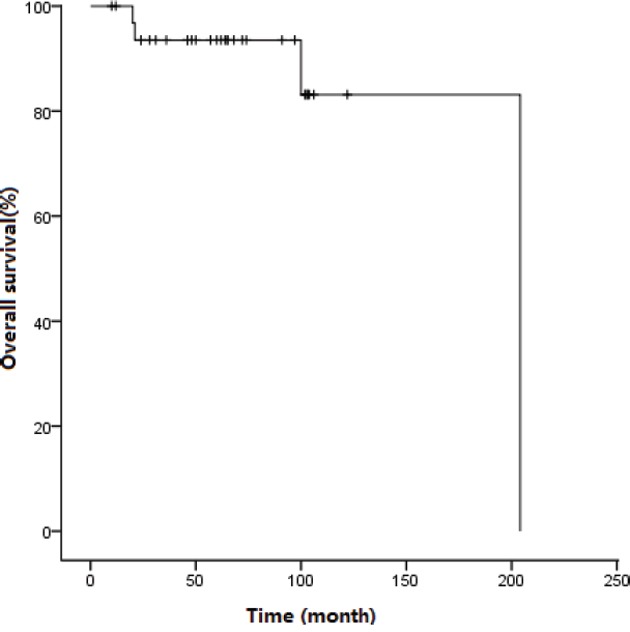
Survival curve for patients treated with imatinib

At the end of the follow up, 29 patients were alive, with a median follow-up of 64 (10-204) months, and the median survival did not reach significance. The 5-year OS was 93.5±4.4% (Figure 2). Four patients (patients 1 to 4) died at a median time of 60.5 (20-204) months after presentation.

## DISCUSSION

The clinical and laboratory features of 33 patients with F/P mutated CEL were retrospectively reported. All 33 patients were male, and the specific reasons leading to this remain unknown. Eosinophil contributed to tissue remodeling and thrombosis via the release of eosinophil granules that contain major proteins and numerous cytokines [14]. The common clinical manifestations reported in the literature included cutaneous, pulmonary, gastrointestinal, cardiac, and neurological abnormalities [15]. Our data indicated the main characteristics of the patients with F/P rearrangement included severe eosinophilia, substantially increased vitamin B12, splenomegaly, anemia, thrombocytopenia, and myelofibrosis. These features were in accordance with myeloproliferative neoplasms (MPN). In addition, the most frequent organ involvement was the spleen, followed by the lung, skin, liver, and heart. The cutaneous, pulmonary, and gastrointestinal symptoms were typically unspecific. The cardiac involvement presented with clear discomfort, and the most common damages included endomyocardial fibrosis and valvular abnormalities diagnosed via echocardiography and MRI. The neurological damage manifested as stroke and peripheral neuropathy. Moreover, thromboembolism should be a matter of vigilance, especially the extensive lower extremity venous thrombosis and pulmonary embolism. These serious complications typically require a multidisciplinary group of clinicians.

The benefit of imatinib in F/P fusion gene positive CEL has since been confirmed in numerous studies, and imatinib is recommended as a clear first-line therapy in patients with F/P-positive CEL [7–11]. One hundred mg/day is sufficient to induce a CMR and histological response in most patients with an F/P fusion gene, and 100-200 mg/week may be sufficient to maintain a durable remission in some patients. In this 33 F/P fusion gene positive CEL patient cohort, most of whom received 100 mg/day as the initial dose, thirty-two (97%) patients achieved a CMR, and the median time was 3 months, which was shorter than the 12 months previously reported by Helbig et al [9] but similar to recent results from the Mayo clinic [16] and another French group [17]. This difference may be explained by a shorter period of daily imatinib treatment in the former study. In our study, most patients decreased the dose when a CMR was achieved; however, in Helbig et al, the dose was typically reduced at the time of a CHR. Our results also demonstrated that 100 mg/week effectively maintains the response, which is consistent with other reports.

Limited information regarding the discontinuation of imatinib treatment for F/P fusion gene positive CEL has been reported. Although in-depth and durable molecular responses occur with imatinib, drug discontinuation may lead to relapse. One early study included 4 cases administered imatinib for a median of 9.5 months (range 7-14); all of the cases relapsed, and the median relapse time was 25.5 months (range 19-31) following imatinib discontinuation [10]. Recently, a group of French researchers reported 11 cases of discontinuation; 6 cases underwent a median imatinib treatment of 38.3 months (range 2-99), and relapse occurred from 1 to 27 months after discontinuation, whereas the remaining 5 cases received imatinib treatment for a median of 30.2 months (range 21.3-45.5) and maintained a CHR or CMR 31 months (range 9-88) after discontinuation [17]. In an isolated case report, 2 cases discontinued imatinib after a 5-year CMR duration and maintained a CMR 24 months after discontinuation [18]. In our group, 8 cases stopped oral imatinib after achieving a CMR; 4 cases discontinued imatinib with a median treatment of 17.5 months (range 4.5-24) and subsequently relapsed at a median follow up of 8.5 months (range 2-48), and 1 patient (patient 2) maintained a CMR duration of 48 months after discontinuation. The remaining 4 cases maintained a CHR or CMR 47 months (range 2-74) after discontinuation; these patients had received imatinib for a median of 19 months (range 8-24). Our findings suggested that some patients may maintain long-term remission following the termination of therapy at the CMR status; however, other patients require a long-term treatment to maintain a durable remission. To date, the specific reasons for this difference remain unknown; we are using exomic- and whole genome sequencing in our patients to identify biomarkers for imatinib termination in F/P fusion gene positive CEL. At present, evidence indicates that cessation of imatinib make most remission patients lose CMR, although a few patients acquire long-term remission after discontinuation, so we suggest imatinib treatment should be continued after achieving a CMR, and discontinuation be only used on perspective clinical trial. The patients who have withdrawn imatinib should monitor molecular changes closely, and restart imatinib treatment as soon as possible when fusion gene is detected again.

Evidence indicates that imatinib resistance has been a rare event in patients with F/P fusion gene and is typically related to disease progression [19–23]. A T674I mutation in the ATP-binding region of PDGFRA was the most common imatinib-resistant mutation and frequently occurred in the acceleration or blast phase. Moreover, nilotinib and sorafenib had limited clinical activity in the T674I mutation. In our resistant cohorts, 3 patients were determined to have a T674I resistant mutation at the chronic and blast phases, respectively. One patient exhibited primary imatinib resistance in the chronic phase. The other 2 patients acquired imatinib resistance at relapse during the drug withdrawal period. Drug withdrawal may be a risk factor for imatinib resistance. All three patients died after drug resistance; however, a combination of chemotherapy regiments was used. Thus, hematopoietic stem cell transplantation should be the first choice for these patients for a longer survival. Moreover, Some novel TKIs like ponatinib [24] or HSP90 inhibitor [25] should be investigated to against T674I mutation in clinical.

In conclusion, the current findings demonstrated the overall clinical and laboratory features of Chinese patients with F/P mutated CEL and concurrently indicated the outcome of imatinib treatment. To the best of our knowledge, this series is the largest F/P mutated CEL dataset to date from Asia. In the TKI era, the prognosis of F/P mutated CEL has been substantially improved. In our groups, the 5-year OS was 93.5%, which was similar to a previous report in a western population [16]. In the future, the important issues include the identification of patients that may safely discontinue imatinib and how to overcome drug resistance.

## MATERIALS AND METHODS

### Study population

Thirty-three consecutive patients with F/P mutated CEL were diagnosed or re-diagnosed according to the 2008 revised WHO classification criteria [3]. The patients treated with imatinib between August 2006 and October 2014 in the Institute of Hematology and Blood Diseases Hospital, Chinese Academy of Medical Sciences (CAMS) and Peking Union Medical College (PUMC) were included in this study. All patients were male with a median age of 35 (18-73) years. This study protocol was approved by the Ethical Committee of the Institute of Hematology, CAMS and PUMC following the ethical principles of the Declaration of Helsinki.

### Baseline evaluation

In general, a complete history and physical examination were carefully evaluated in all patients, and laboratory tests were performed at baseline, including a complete blood count, multiple stool ova and parasite testing, chemistries, serum total IgE, vitamin B12, bone marrow (BM) morphology, conventional cytogenetics, F/P fusion gene (by nested polymerase chain reaction (PCR)), and T cell receptor (TCR) gene rearrangement PCR. Electrocardiogram, echocardiography, chest radiography, and abdominal ultrasound were also performed at baseline to determine organ involvement. Tissue biopsies were obtained when clinically indicated. The PDGFRA/B and FGFR1 rearrangement at baseline were analyzed via fluorescence in situ hybridization (FISH). More than grade 2 was recognized as myelofibrosis according to the European consensus on grading bone marrow fibrosis [13]. Splenomegaly and hepatomegaly were present when the organ could be touched below the rib edge. The normal values of serum IgE and vitamin B12 were <165.3 IU/mL and <800 pmol/L, respectively.

### Detection of F/P fusion gene at diagnosis and for monitoring minimal residual disease

RNA extraction from BM samples and cDNA synthesis were conducted using standard procedures after the isolation of mononuclear cells following red cell lysis. Nested RT-PCR for the detection of the F/P fusion gene was performed as previously described [1]. The following primers were used: FIP1L1-F1, 5′ acctggtgctgatctttctgat; PDGFRA-R1, 5′ tgagagcttgtttttcactgga; FIP1L1-F2, 5′ aaagaggatacgaatgggacttg; and PDGFRA-R2, 5′ gggaccggcttaatccatag. All amplifications were performed for 35 cycles at a 65°C annealing temperature.

### Treatment regimen and response assessment

For the 33 patients, the initial dose was 100 mg/day in 30 patients and 200 mg/day in the remaining 3 patients. The maintenance dose depended on the response condition and patient willingness. A complete hematologic remission (CHR) was defined as a decrease in the absolute eosinophil count (AEC) to the normal range (0-0.5×10^9^/L) with a normal hemoglobin (Hb) value (≥110 g/L) and blood platelet count (BPC) (≥100×10^9^/L)[15]. Partial hematological remission (PHR) was defined as a decrease in the eosinophil count to less than 50% of the baseline value, but not in the normal range. No hematological remission (NR) was defined as a stable or increasing eosinophil count. Complete molecular remission (CMR) was defined as a negative nested RT-PCR for F/P transcripts. The national cancer institute common terminology criterion for adverse events (NCICTCAE) version 3.0 was utilized for side effect grading.

### Statistical analysis

Nonparametric comparisons of group means were conducted using Mann-Whitney U tests. Proportions were compared using Chi-squared tests. The overall survival (OS) was analyzed using the Kaplan-Meier method, and the survival times were measured from the date of diagnosis to the date of death or loss during follow-up. A *P*-value less than 0.05 was set as the level of statistical significance. All analyses were performed using SPSS software (version 13.0; SPSS, Inc., Chicago, IL). Data were censored at August 20, 2015.
